# Perspective: The role of mechanobiology in the etiology of brain metastasis

**DOI:** 10.1063/1.5024394

**Published:** 2018-05-08

**Authors:** Kandice Tanner

**Affiliations:** Laboratory of Cell Biology, Center for Cancer Research, National Cancer Institute, National Institutes of Health, Bethesda, Maryland 20892, USA

## Abstract

Tumor latency and dormancy are obstacles to effective cancer treatment. In brain metastases, emergence of a lesion can occur at varying intervals from diagnosis and in some cases following successful treatment of the primary tumor. Genetic factors that drive brain metastases have been identified, such as those involved in cell adhesion, signaling, extravasation, and metabolism. From this wealth of knowledge, vexing questions still remain; why is there a difference in strategy to facilitate outgrowth and why is there a difference in latency? One missing link may be the role of tissue biophysics of the brain microenvironment in infiltrating cells. Here, I discuss the mechanical cues that may influence disseminated tumor cells in the brain, as a function of age and disease. I further discuss *in vitro* and *in vivo* preclinical models such as 3D culture systems and zebrafish to study the role of the mechanical environment in brain metastasis in an effort of providing novel targeted therapeutics.

## INTRODUCTION

Solid cancers often show preferential organ colonization in line with Paget's original seed-soil hypothesis, based on his examination of breast cancer patients at autopsy.^1,2^ Paget likened tumor cells to “seeds” and the organ of eventual secondary lesion as the “soil.” Paget hypothesized that the establishment of a *de novo* lesion is achieved only if there is compatibility between seed and soil.^1^ For example, ocular melanoma has been shown to preferentially metastasize to the liver, whereas cutaneous melanoma and lung and breast cancers share a common metastatic site in the brain.^2–4^ Metastatic brain lesions account for ∼90% of all central nervous system (CNS) tumors, outnumbering primary brain tumors at a factor of ∼10:1.^5,6^ Of all sites of organ colonization, brain metastases are associated with the worst prognosis, with a median survival of less than a year on average, coupled with a reduced quality of life due to associated physical and cognitive deficits.^7,8^ Despite recent improvements in the treatment of systemic disease and associated brain metastases, the median survival of patients with metastatic brain lesions is approximately 7–16 months from diagnosis.^5–7^ Therefore, understanding (1) how cells target specific organs, (2) whether differences exist in this targeting, and (3) factors critical to cell survival following dissemination is also important for developing optimal treatments for metastatic and resistant tumors.

Tumor latency and dormancy remain the most challenging aspect of cancer dynamics and thus play a role in the lack of appropriately targeted therapies. Specifically in brain metastases, emergence of a lesion can occur at varying latencies from diagnosis and in some cases following successful treatment of the primary insult.^7,9^ Specifically, patients with receptor tyrosine kinase ERBB2+ (also known as HER2+) breast cancer have exhibited elevated incidences of metastastic lesions in the brain.^7^ This tumor type can result in latent disseminated cells re-emerging as aggressive brain cancer, as late as 20 years following initial diagnosis.^2,7,9^ In contrast, 25%–30% of non-small cell lung cancer (NSCLC) patients can present with brain metastases at diagnosis.^10,11^ These timing differences in brain metastatic disease are also observed for other solid tumors that have tendencies to migrate to the brain.^2–4,7,12^ Why is there a difference in latencies between these cancer types? Is there a difference in the “soil” of the brain microenvironment that renders one dormant while permissive for outgrowth in the other? What might change in this environment to drive emergence from dormancy after many decades?

In the last decade, numerous studies have illuminated the importance of the continuous dynamic and reciprocal relationship between cells and the microenvironment. These studies have detailed the ability of mechanical tissue properties, including the geometry, topography, and elasticity of the extracellular matrix (ECM), to influence cell fate decisions.^13–16^ One missing clue may be the role of brain microenvironmental tissue biophysics in infiltrative cells. Here, I focus on biophysical cues that may influence outgrowth of metastatic lesions in the brain. This perspective focuses on the use of 3D culture models and alternative pre-clinical models such as zebrafish to recapitulate human disease. These platforms are extremely powerful in discerning the role of tissue biophysics, in an effort of better understanding the etiology of organ specific metastases and ultimately improve therapeutic options.

## BACKGROUND—HOW DO CELLS COLONIZE THE BRAIN?

The first step of dissemination along the metastatic cascade involves escape from the primary site with the entrance of cells to a drainage system, either the lymphatic or vascular system.^3,4^ Seminal work in the 1970s found that while approximately 3–4 × 10^6^ cancer cells can enter the bloodstream per gram of tumor on a given day, only about 0.01% of these cells survive the passage. Many of these cells are unable to endure the environmental stresses associated with the journey.^4,17^ Yet, those that do survive will invade and persist in distant organs, eventually resulting in secondary disease. Brain metastases are thought to arise largely due to hematogenous dissemination.^9^ However, dissemination throughout the leptomeninges can also be achieved by transit from existing lesions in the brain, venous plexus, nerves, perineural/perivascular lymphatics, and the choroid plexus.^7^ After transit, these tumor cells often arrest in the dense brain capillary network.^7,9^ After initial arrest in the capillary bed, tumor cells may either remain as quiescent cells or actively proliferate to establish a secondary lesion.^2,3,7^ Gross examination reveals that regional distribution of metastatic lesions correlates with the regional blood flow and brain volume.^18^ Approximately 80% of lesions are found in cerebral hemispheres, 15% in the cerebellum, and the remainder in the brain stem.^18^ These cells continue to face a myriad of challenges before they can override the host organ to form clinically relevant lesions.

Organ colonization is the most complex and rate-limiting phase of the metastatic process.^2,9^ The term “colonization” here defines the balance of tumor cell proliferation, apoptosis, and quiescence in the formation of a metastatic lesion.^2,9^ Both growth and continued single cell survival necessitate access to metabolites and oxygen.^2,3,7^ Within the brain, two strategies have been identified to facilitate tumor outgrowth: avascular or neo-angiogenic proliferation in the absence or the presence of newly created blood vessels, respectively.^7,19–21^ Avascular growth occurs when tumor cells co-opt existing vessels, proliferating and migrating along the abluminal surfaces.^20,21^ The latter strategy is the one in which tumor cells that have invaded tissue parenchyma create new vasculature to enable continued proliferation, invasion, and successful expansion beyond a tumor volume of ∼1–2 mm^3^.^9,19^ Jain *et al.* present an excellent review on the cytokines and cellular mechanisms that govern angiogenesis in the brain for further examination.^22^

## BACKGROUND—DEFINING THE BRAIN MICROENVIRONMENT

After initial arrest in the capillary bed, tumor cells encounter the endothelial layer and its basement membrane amongst the blood brain barrier (BBB).^13,14^ The Young's modulus of this endothelial barrier approaches ∼5 kPa for *in vitro* models.^23,24^ Thus, the BBB provides the first mechanical barrier before entry. During extravasation, tumor cells show extensive deformation of the cell and its nucleus in order to infiltrate the tissue.^25,26^ Recent work has shown that cancer cell migration in tight spaces induces rupture of the nuclear envelope and compromises DNA integrity.^25,26^ Thus, identifying the difference between pre- and post-extravasation of the infiltrated cells in itself is non-trivial! However, below I focus on the cues that may influence cell post-extravasation.

**FIG. 1.**
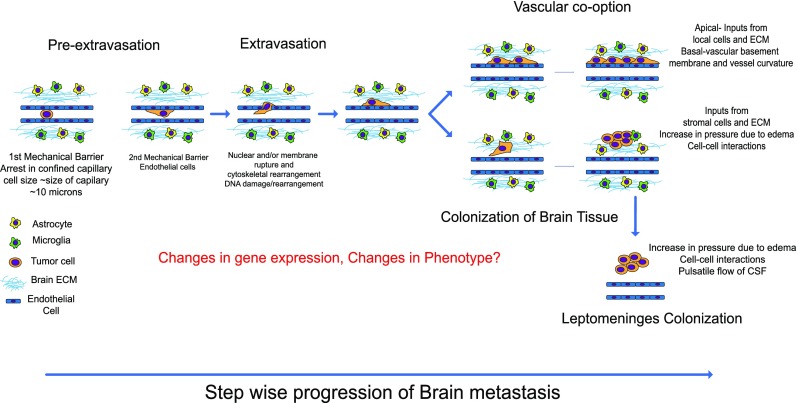
Schematic of the metastatic cascade depicting the tumor cell cross-talk with the brain microenvironment from the time of arrest in brain capillaries following post extravasation outgrowth strategies that have been observed in clinical and animal models.

After extravasation, these cells may colonize three different microenvironments: the brain parenchyma, perivascular spaces, and leptomeningeal niches (Fig. 1).^7,9^ Tumor cells co-opt existing blood vessels, where they adhere to the vascular basement membrane via β1 integrin, proliferate, move, and progressively invade into the brain parenchyma.^20,21^ Additionally, these tumor cells not only maintain contact with the pericytes that line the vessels but also interact with glial cells, astrocytes, and ECM. The brain's ECM composition is also highly specialized, where one of the major components is hyaluronic acid (HA), a non-sulfated glycosaminoglycan.^7,27,28^ HA occupies a large fraction of the extracellular volume of the brain. However, it is relatively soft.^29^ In addition to HA, brain ECM contains other glycosaminoglycans such as heparin sulfate, a number of chondroitin sulfate proteoglycans of the lectican family such as aggrecan, neurocan, and versican and proteins such as Tenascin-C and thrombospondin. In contrast, there is a relatively low amount of fibrillar proteins such as collagen type I, fibronectin, and vitronectin within the ECM microenvironment.^28,30^ These in addition to basement membrane proteins, such as laminin, are largely restricted to the vascular and perivascular spaces in the brain.^7,30^ In some cases, after the tumor cells have breached the BBB, they directly invade into the brain parenchyma with very little interaction with existing vessels.^7,22^ Alternatively, tumor cells proliferate, generate their own vasculature, and displace brain tissue, driving a chronic inflammatory response, where both resident and recruited immune cells become activated at the site of the lesion.^7,22,31^ Finally, tumor cells may occupy the interstitial spaces such as between hemispheres containing the cerebrospinal fluid (CSF) and ependymal linings of the brain.^18^ Within this environment, tumor cells are exposed to the pulsatile flow of the CSF, which assists with waste turnover, varying electrolyte levels, and patrolling leukocytes.^32,33^

Within the dynamic nature of the microenvironment, physical cues that cells receive are distinct.^14,34^ For example, in the case of vascular co-option, the topography of blood vessels provides signals to surrounding tumor cells that invade and proliferate within the brain parenchyma, which can all be influenced by the local tissue mechanics. The role of tissue mechanics in cancers that originate in the brain has been studied; such as an increase in tissue stiffness is observed in high grade glioma.^27,35^ However, the effect of the mechanical environment on infiltrating tumor cells remains largely unknown.

## MECHANOBIOLOGY IN THE TUMOR MICROENVIRONMENT

Mechanobiology describes how physical properties can regulate sub-cellular signaling cascades where integration of these signals then tunes biochemical and genomic pathways to affect cell fate decisions. Specifically, the physical properties of the microenvironment, such as tissue mechanics, shear forces due to fluid flow, and surface topography, have been shown to modulate gene expression and phenotype.^15,16,36,37^ These properties then in turn can direct proliferation, motility, apoptosis, or quiescence. Consequently, cells interpret these environmental cues via protrusions such as invadopodia, podosomes, filopodia, or lamellipodia.^38–41^ These protrusions are critical not only in invasion, adhesion, and locomotion but also in degradation of extracellular matrices.^38,39,41^ These micron scale features contain specialized machinery to detect and interact with specific ligands depending on the types of signaling.^38,41^ For single cells, quiescence may be regulated by apoptotic and proliferative signals initiated at the surface of protrusions.^42,43^ Moreover, cargo can be trafficked in secreted vesicles or released as cytokines, proteases, and hormones directly to and from adjacent cells within the milieu.^44,45^ The sum of these interactions contributes to the physical remodeling of the microenvironment. While these protrusions vary in size and shape (containing cytoskeletal structures such as microtubules and actin stress fiber bundles), the typical protrusion is about 1 *μ*m thick.^38,41^

In addition to these protrusions, specialized adhesion complexes, such as focal adhesions and hemidesmosomes, allow cells to sense physical properties such as tissue mechanics. Cells generate traction forces and transmit these forces to neighboring cells and local ECM.^40,46^ In the event that the emergent phenotype allows for active proliferation, then as the cluster grows, homotypic interactions with neighboring cells further influence cell fate decisions. In mammalian systems, microenvironmental cues such as tumor-stromal crosstalk are important regulators of disseminated tumor outgrowth.^7,13,25^ Thus, heterotypic physical interactions of tumor cells with stromal cells (such as resident cells in the organ or resident/recruited immune cells) also contribute to the mechanical microenvironment.^14,15^

The mammalian brain is soft compared to other tissues.^47,48^ Brain mechanics have been elucidated using modalities such as micron scale based tip atomic force microscopy (AFM), Magnetic Resonance Elastography (MRE) and standard shear rheometry, which demonstrate the dynamic nature of its nonlinear viscoelastic material.^49–52^ Based on the methodology, timescale, and applied strain, the shear or Young modulus can range from 0.04–20 kPa.^29,52^ Interestingly, this range is comparable to the Young's moduli measured at the invasive front of human breast tumors and the adjacent normal tissue.^53^ Thus, the brain may more closely match the tissue mechanics of the primary organ. Goriely *et al.* provide an extended overview of the challenges and opportunities involved in measuring brain mechanics at different lengths and timescales.^54^ Brain resident cells such as neurons, astrocytes, and microglia within a specialized ECM contribute to the viscoelasticity of the brain.^29,55,56^ Within this environment, cells are also exposed to the differential flows of CSF, blood, and interstitial fluid.^54,57,58^ Here, we focus on the biophysical cues that may influence cell behavior after cells have successfully infiltrated the brain.

## WHAT PHYSICAL CUES MAY INFLUENCE EARLY COLONIZATION IN THE BRAIN?

### Post-extravasation—Vascular co-option

As tumor cells breach the endothelial barrier, a single cell or clusters of cells line the interface of the vascular basement membrane of the blood vessels. Vessels within the brain are arboreal in nature where bifurcations and variations in architecture allow for energy requirements of the brain. In this geometrical configuration, surface cues from the vessel in concert with the local viscoelasticity of the brain tissue may influence initial cell fate decisions. Specifically, for disordered regions (defined as high curvature, non-linear areas) with respect to the size of the cells, topographical cues may induce changes in gene expression. Conversely, the local viscoelasticity of the tissue may also provide a cue that modulates proliferation and expansion into tissue or along the vasculature. Furthermore, within regions such as the cerebellum and cerebrum, gray and white matter also demonstrates regional heterogeneity in tissue mechanics. The mechanical properties of gray and white matter have been measured to be distinct, where some measurements indicate that white matter is stiffer than gray matter, whereas the reverse is reported for other measurements.^59^ Using Magnetic Resonance Elastography, shear stiffness of gray matter varies from ∼1–5 kPa, whereas comparable measurements show values of ∼1–15 kPa for white matter.^60–64^ Yet, it remains to be determined how these cells interpret each of the surrounding physical cues. Is there a hierarchy where one property, topography vs. mechanic signal, overrides the other? Where would such a distinction be necessary and in what circumstance?

### Post-extravasation—Brain parenchyma colonization

Tumor cells may directly invade into brain parenchyma. At the single cell level, tissue mechanics may be the dominating cue. A bi-directional interaction between the tumor cell and the tissue exists, whereby contractile cells directly secrete cytokines and *de novo* extracellular matrix components that in turn dynamically tune local properties to either establish single cell quiescence or enable further outgrowth.^65^ In the event of proliferation, imaging studies reveal that as cells grow and displace surrounding tissue, concomitant formation of *de novo* tumor vessels and several other biomechanical changes occur.^20–22^ Specific examples of biomechanical modifications include multicellular contractile forces, induced interstitial pressure, and edema due to impaired circulation of underlying brain vasculature, which all drive the evolution of the mechanical milieu.^20–22^ In the incidence of gliomas, interstitial fluid pressure (IFP) ∼9.1 ± 2.1 mmHg, whereas in normal brain tissue, IFP is ∼0 mmHg in the steady state.^66^ The increase in fluidic pressure activates similar mechanotransduction pathways such as ECM stiffening.^67^

In the case of quiescence, single cells may establish a mechanical equilibrium with their local environment, in an effort of surviving in a dormant state. Yet, these quiescent cells may not be able to overcome the mechanical constraints required for tumor outgrowth. Nevertheless, if this equilibrium is disturbed, these cells may either die or become aggressive and proliferate to form lesions.

### Post-extravasation—Leptomeningeal colonization

Leptomeningeal metastasis is rarely the first site of metastasis, but instead observed in patients with widely metastatic and advanced cancer.^68,69^ Leptomeningeal metastastic spread remains a clinical challenge, as patients often succumb within 8 weeks if the tumor is left untreated.^69^ Unlike the environment found in other regions, invading tumor cells do not interact with diverse stromal cells but remain largely as single cell or multicellular spheroid suspensions within the cerebrospinal fluid (CSF). These tumor outgrowths may attach to the pia mater, a thin mesenchymal tissue layer that coats the spinal cord and roots.^69^ Within this acellular environment, tumor cells are exposed to the shear stresses of ∼100 Pa and ∼0.4–0.6 kPa due to the pulsatile flow of the CSF, as measured in canines and predicted by computational fluid dynamics in humans, respectively.^70,71^ The dynamic nature of CSF pulsatile flow is multifactorial and has been directly correlated with changes in cardiac output.^70,71^

## BRAIN METASTASIS VS PRIMARY CENTRAL NERVOUS SYSTEM (CNS) TUMORS

As observed in brain metastasis, primary CNS tumors are sensitive to the physical properties of the brain microenvironment. Primary CNS tumors such as Glioblastoma Multiforme (GBM) respond to mechanical signals, where increased interstitial pressure drives GBM tumor growth and invasion.^72,73^ Moreover, motile GBM cells stiffen their surrounding matrix, which, in turn, increases motility.^74^ As primary CNS tumors approach diameters greater than 1–2 mm within the brain parenchyma, mechanical properties are modulated. These tumor induced changes are due to compromised structural and functional adaptations promoting neovascularization and/or edema and/or tissue degradation.^22^ Primary CNS tumors are stiffer than normal brain tissue, where a steady state modulus of ∼0.2–16 kPa has been measured for freshly excised human tumors.^75^ However, there is one major distinction between primary CNS tumors and disseminated cells originating from breast, lung, and skin tumors. The latter experience a suite of mechanical cues such as shear stresses, interstitial pressure, and vascular constrictions along the metastatic cascade, which may assist and drive the emergence of brain metastasis.^13,14,76^ Using human bone-targeting, lung-targeting, and non-metastatic breast tumor cells, Kostic *et al.* demonstrated clones cultured on substrates that match the stiffness of the preferential site of colonization, yielding increased proliferation and migration occurrences.^77^ These clones contained specific gene signatures that determine tropism in murine models. Genes such as C3, ST6GALNAC5, and members of the class of Serpins have been identified as important for homing to the brain.^77–80^ Interestingly, most of these genes are distinct from those that are overexpressed in primary CNS tumors except for epidermal growth factor receptor (EGFR) which is often amplified or mutated in GBM.^79,81^ Thus, it may be that the transition of cells from the circulation from a primary organ with a given physical microenvironment to the parenchyma of the brain, which houses different microenvironmental properties, may also be a key regulator of the marked differences in tumor growth, vascularization/angiogenesis, and/or dormancy patterns in primary vs. metastatic lesions.

## TUMOR LATENCY—TO GROW OR NOT TO GROW?

The brain is often a site of relapsed metastatic diseases. While these relapses are only seen in a subset of cancers, recurrent disease diagnosis is often several decades post primary diagnosis despite initial eradication of disease at the primary site. Recent studies suggest that a subset of tumor cells disseminate at early stages of growth from the primary site and remain as quiescent cells in a distant organ. Aguirre-Ghiso and colleagues present an overview on the factors that may drive the establishment of dormancy.^82,83^ Here, I focus on what may drive an exit of tumor cells from dormant to active within the brain. Murine models have shown that dormant cells become proliferative when fibrosis is induced in the lungs via the Src-beta 1-FAK signaling cascade.^43,84^ However, an additional reason may be that a fibrotic lung is stiffer than a normal lung.^85^

Alterations in brain mechanics are observed in many pathological states associated with disease and those due to traumatic injury.^54^ Several studies have linked individualized cell mechanics to alterations in brain extracellular matrices and loss of neurons, which may collectively or individually modulate the viscoelastic properties of the tissue.^29^ The mechanical properties of the brain can be influenced in part due to the age and presence of non-cancer related disease and those due to local and/or systemic treatment. Within this transformed environment, the emergent changes may re-awaken quiescent tumor cells that have been resident within the brain for several years.

### Changes in brain tissue mechanics due to normal aging

Age is considered a risk factor for several different solid tumors.^86^ Loss of tissue architecture concomitant with changes in the cellular composition is observed in the aging brain.^87,88^ Specifically, a progressive loss of neurons and oligodendrocytes results in a rough loss in the volume of ∼3.7 cm^3^/year.^52^ This tissue degradation and the associated decline in cognitive function are distinct from effects due to neurodegenerative disease.^88^ These age-related changes may also change the overall tissue mechanics of all cells spanning from the micron scale (cells) to the millimeter scale (tissue). Examination of rheological properties on the scale of millimeters of freshly excised human brains revealed a difference in organ mechanics as a function of age.^52^ Moreover, the brainstem was found to be approximately 2–3 times stiffer than both gray and white matter.^52^ Non-invasive mapping of brain mechanics can be obtained using magnetic resonance elastography (MRE).^89,90^ This technique specifically detects changes in brain viscoelasticity by combining standard MRI with acoustic waves.^89,90^ It has the advantage of longitudinal studies of the same patient performed repeatedly to deduce changes in mechanical properties as a function of age.^55,89^ In a study that examined patients between the ages of 18 and 72 years old, where the shear waves were applied by a superposition of four harmonic frequencies 25, 37.5, 50, and 62.5 Hz, global average shear storage and loss moduli of the whole brain were determined using MRE ranging from 1.64 to 2.58 kPa and 0.8 to 1.31 kPa, respectively.^90^ These age dependent changes in the viscoelastic properties of the brain have been confirmed in porcine, bovine, and rodent animal models.^89^

### Changes in brain tissue mechanics due to disease

Loss of brain elasticity not only is associated with normal aging but can also be influenced by the presence of diseases such as Multiple sclerosis (MS) and Alzheimer's disease (AD), Amyotrophic lateral sclerosis (ALS) or due to repeated trauma.^91–94^ MS is a demyelinating disease in which the insulating covers (myelin) of nerve cells in the brain and spinal cord become damaged, resulting in plaque deposited lesions within the white matter.^95^ Patients with AD show an accumulation of extracellular amyloid plaques, intracellular neurofibrillary tangles, and neurodegeneration, whereas in ALS, degeneration of the corticospinal tract (CST) results in significant neurologic disabilities.^96,97^ Specifically, in studies comparing patients suffering from symptomatic AD with age matched healthy patient controls, the shear elastic moduli ranged from 1.96 to 2.29 kPa and 2.17 to 2.62 kPa.^91,92^ However, while the overall stiffness of the global brain may be diminished, the sum may not be indicative of the local microscale heterogeneities. For example, amyloid fibrils themselves are six orders of magnitude greater in stiffness than neurons and glial cells.^56,98^ These findings give credence to the plausibility of mechanical properties of the metastatic niche playing a key role in successful colonization of distant organs.

## ENGINEERING THE BRAIN—PRE-CLINICAL MODELS OF THE MICROENVIRONMENT

**FIG. 2.**
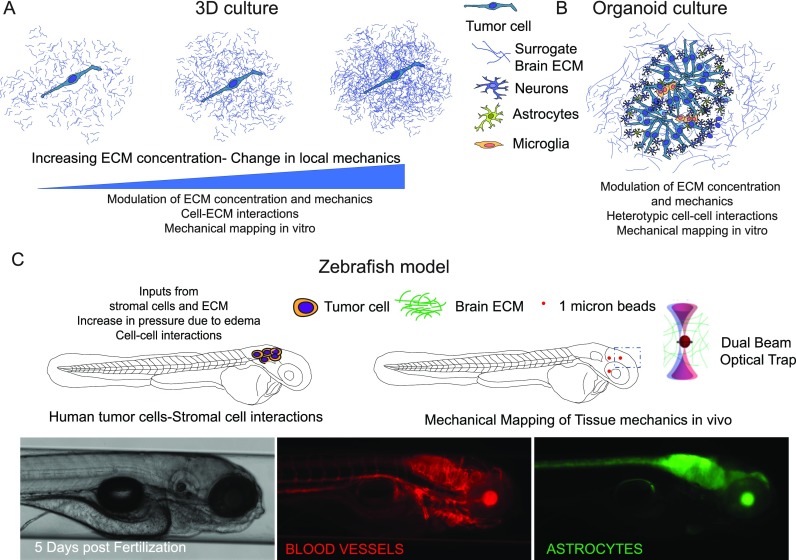
Preclinical models that recapitulate the brain microenvironment. (a) 3D culture of single cells embedded in HA gels with different amounts of crosslinkers to study single cell-ECM interactions. (b) Schematic of brain organoid culture where colors indicate the different lineages that can be derived from induced pluripotent stem cells (iPSCs) within brain ECM mimetics to study heterotypic cell interactions in 3D. (c) Larval Zebrafish model—schematic shows injected tumor cells in the brain of the larval fish and ability to map the mechanical properties of the brain using optical trap based active microrheology. Micrographs show a transgenic fish where both blood vessel and astrocytes can be visualized to study human cell-brain stroma interactions.

The temporal gap between infiltration to distant organs and the ability to colonize and form macrometastases within the brain remain difficult to access and obviously is *a priori* in secondary brain tumor patients. Moreover, linking the mechanical properties to the etiology of metastasis is difficult. Further investigations are limited by non-invasive techniques for mechanical mapping at the microscale and the paucity of samples available at autopsy. Below, these culture systems are amenable to tailoring the *in vivo* mechanical cues, thus recapitulating the heterotypic interactions between cells and the microenvironment **(**Fig. 2).

## 3D CULTURE MODELS

3D culture models have now become routine to recapitulate *in vivo* microenvironments in a modular manner. They also allow for mechanical mapping of the 3D environment and visualization of tumor dynamics from minutes to days.^72,99–106^ Specifically, one can study cell-ECM and cell-cell interactions in the presence or the absence of physiological shear forces by incorporating microfluidics. These culture studies have been powerful to study proliferation, migration, and drug efficacy in a controlled manner for both primary brain cancer and tumor cells that metastasize to the brain. In these studies, cells are embedded in 3D hydrogels comprised of HA and exposed to an isotropic distribution of ECM.^107–110^ These systems allow for the study of single or multicellular aggregates.^108–110^ Spheroids can be generated due to clonal expansion, spontaneous aggregation, liquid overlay cultures, or using gyratory bioreactors. Hybrid matrices may also be employed to allow the examination of brain specific ECMs such as HA, Tenascin C, Laminin, and those that may be secreted by the tumor cells such as Fibronectin and Collagen type 1.^108,111^ Additionally, mechanical properties can be modulated by cross-linking enzymes.^107,109,110^ Architectural complexity such as fibers can be incorporated into 3D amorphous hydrogels using self-assembly of magnetic colloidal particles functionalized with ECM proteins.^111^ These platforms are amenable to incorporation of additional brain specific cells such as neurons, astrocytes, brain endothelial cells, and microglia. Moreover, brain specific structures can also be achieved such as the very complex BBB. Specifically, using commercially available transwell culture systems and microfluidic platforms, the physical characteristics of the BBB including tight junctions and restrict transfer of large molecules as measured by transendothelial electrical resistance and permeability, have all been achieved.^112,113^ Using these systems, one can directly test both the chemical and physical effect on tumor cell motility and invasion. Recently, Shumakovich *et al.* determined that astrocyte-secreted factors alter migration and morphology of metastatic breast tumor cells.^114^ This effect was in part regulated by the local mechanical microenvironment.^114^ Co-culture can also be done directly with brain tissue, where tumor cells are cultured *ex vivo* on thick slices of brain.^80,115^ Incorporation of microfluidics emulates *in vivo* conditions such as perfusion, physiologically relevant shear forces, and nutrient and waste exchange within these tissues. Additional complexities such as topography of interaction, degree of contact, and the number of cells that can be co-cultured can be achieved using a combination of microfluidics and micropatterning.^116^

## 3D-BRAIN ORGANOID CULTURES

A recent explosion in 3D organoid cultures, due in part to technological advances, has allowed for *in vitro* recapitulation of complex *in vivo* 3D structures of tissues that are often optically inaccessible in humans.^117–119^ To differentiate between co-culture studies aforementioned, an organoid is defined as a 3D structure grown from stem cells where organ-specific cell types self-organize through cell sorting and spatially restricted lineage commitment.^118^ Seminal work from the Sasai lab first demonstrated the feasibility of generating complex tissue *in vitro.*^120–124^ This work showed that not only it is possible to derive differentiated cell types from stem cells but also biologically relevant spatial patterning and morphogenesis can be achieved in a dish. Specifically, they developed methods that controlled both the size of 3D aggregates of human pluripotent cells and chemical cues to generate brain structures such as the retina and pituitary “in a dish.”^121–124^ Building on these findings, cerebral organoids can now be grown where the tissue can realize sizes of up to a few millimeters when grown in a spinning bioreactor.^125^ Within these structures, additional cell types and architectures are observed such as the retina, dorsal cortex, ventral forebrain, midbrain-hind-brain boundary, choroid plexus, and hippocampus.^119^ One concern is the ability to have matured cells that show cell specific markers, organization, and functionality. Recent work demonstrated the feasibility of maintaining brain organoids in culture over extended periods (over 9 months) with the formation of dendritic spines and spontaneously active neuronal networks which had not been previously achieved.^126^ These systems have been powerful *in vitro* systems for the study of developmental defects associated with neural progenitor dysfunction and zika virus infections.^127,128^ These studies were largely due in part to the ability to culture pluripotent stem cells *in vitro.*^129^ However, the ability to reprogram somatic cells into induced pluripotent stem cells (iPSCs) that can then be coaxed into specific cell lineages increases the accessibility for brain research. Human brain “assembloids” as described by Pasca in a recent review refer to the incorporation of the iPSC derived brain tissue with other cell types to facilitate cross talk between the central nervous system (CNS) and other tissue.^119^ Yet, it remains to be seen how these organoid studies can be harnessed for organ specific modeling of human brain metastasis or primary brain cancers.

## ALTERNATIVE PRE-CLINICAL MODELS—ZEBRAFISH

Of all the animal models, mice remain the dominant system for the study of human metastatic disease due to the existence of viable immunocompromised strains.^130,131^ However, other animal models such as zebrafish, drosophila, canines, and chick embryos traditionally used for the examination of development can be used to study the link between mechanobiology and cancer.^101,132–137^ In particular, the use of zebrafish for gaining a detailed and dynamic understanding of metastasis is gaining traction in the field of oncology.^138^ The system has been used extensively to study tumor-vasculature, tumor-endothelial interactions, and extravasation in the presence of flow.^139–141^ The availability of transgenic (Tg) zebrafish in which vascular endothelium, astrocytes, and host immune cells endogenously express fluorescent proteins enables detailed studies of host cell-tumor interactions during metastasis.^138,142–144^ Importantly, cytokines associated with tumor progression in murine and human studies are conserved in the zebrafish, and 70% of human genes have at least one zebrafish orthologue.^138,145^ At 72 h post fertilization (hpf), organs such as the heart, brain, and hematopoietic niche (caudal vascular plexus) are relatively developed. At this time, organ specific markers are conserved across vertebrates and share similar extracellular matrix components, thus allowing us for systemic interrogation of metastatic stages.^145^ The zebrafish brain architecture and individual cell and extracellular matrix (ECM) components are also conserved within the mammalian brain.^145^ Excitatory and inhibitory neurotransmitters are highly conserved, including dopaminergic, cholinergic, and GABAergic signaling.^145^ Finally, the developmental program that drives the formation of the architecturally distinct fore, mid, and hindbrain from the embryonic neural tube is conserved between zebrafish and mammals.^145^ Successive collective cell migration and differentiation drive the establishment of specific components of the mature brain such as the cerebellum, medulla thalamus, and tegmentum completely, all with a functional blood brain barrier and choroid plexus.^145^ In addition to tissue architecture, the cell types, astrocytes, neurons, microglia, and oligodendrocytes, and ECMs containing laminin and HA form the specialized brain microenvironment, which have been identified in zebrafish.^145^ This conservation of tissue specificity across vertebrates, in concert with rapid development, makes zebrafish an ideal model for the study of brain metastasis. Recent studies have visualized the interactions between human tumor cells with brain vasculature and brain resident immune cells.^146,147^ Human tumor cells were introduced by direct injection into either the hindbrain or the caudal vein (akin to equal access to all organs such as an intra-cardiac injection into the mice).^146,147^ Specifically, zebrafish macrophages were observed to transmit the cytoplasmic material to human melanoma cells, which then increased tumor cell motility and dissemination.^146^ Also, human breast cancer cells that hone to murine brains can also show increased non-random organ targeting in the zebrafish.^147^ The ease of CRISPR–Cas9-based genome engineering, immune compromised strains, high throughput imaging, and transcriptomics has the potential to provide access to unprecedented details on the earliest stages of tumor cell colonization within the brain.^148–150^ Mechanical characterization of tissue mechanics and hemodynamic forces using optical traps has also been achieved in the fish.^99,101,135^ These studies allow for the direct comparison with 3D culture environments not readily available in murine models.

## CONCLUDING THOUGHTS ON PAGET'S SEED SOIL HYPOTHESIS

Intrinsic differences in genes and signaling pathways that regulate organ specificity have been evaluated using murine models where metastasis is evaluated at the time point of relatively mature lesions.^12,80,151,152^ Priming of the organ niche by tumor derived exosomes and recruited bone marrow derived cells has been implicated in murine studies for colonization of the lungs, liver, and bone marrow. However, the mechanisms that determine how cells transition from the circulation to successfully colonize the “soil” at distant organs are less understood, particularly in the context of the earliest stages of metastasis. As previously described, tumor cells along the metastatic cascade encounter different physical microenvironments during capillary arrest and extravasation into organs.^13,14^ A better evaluation of the relationship between the dynamic nature of primary cells and their metastatic niche is needed to understand non-random organ targeting during metastasis. It remains to be determined if tissue biophysics plays a role in the determination of organ targeting, tumor latency, and/or dormancy. Applying techniques from bioengineering to drive novel pre-clinical platforms will be critical in finally addressing this centuries' old problem. These in concert with preclinical models such as zebrafish may provide insight into why tumor cells home to the brain and the factors that enable survival. These findings may drive improvements in clinical diagnosis, treatment, and prevention in patients who currently have poor disease prognoses.
